# Nutritional Intervention and Physical Activity in Adolescents and Children with Obesity

**DOI:** 10.3390/nu18010101

**Published:** 2025-12-28

**Authors:** Cristina Romero-Blanco, Susana Aznar

**Affiliations:** 1 PAFS Research Group (Promotion of Physical Activity for Health Research Group), Department of Nursing, Physiotherapy and Occupational Therapy, Ciudad Real Faculty of Nursing, University of Castilla-La Mancha, 13071 Ciudad Real, Spain; 2 PAFS Research Group (Promotion of Physical Activity for Health Research Group), Faculty of Sports Sciences, University of Castilla-La Mancha, 45071 Toledo, Spain; susana.aznar@uclm.es

## 1. Recent Advances in the Field

The scientific evidence on nutrition, lifestyle behaviours, and child and adolescent health highlights a reality increasingly shared at the global level: dietary patterns, physical activity levels, sleep habits, and the use of digital technologies are undergoing rapid and interconnected changes that carry significant implications for the health of children and young people [1,2,3]. In this context, recent research has enabled substantial advances in the understanding of the determinants of overweight, energy balance, and the adoption of healthy behaviours during critical stages of development [4,5].

### 1.1. New Perspectives on Dietary Behaviours

Scientific evidence has consolidated the role of factors such as breakfast quality, meal structure, and exposure to ultra-processed foods in shaping children’s health [6,7]. Likewise, considerable evidence demonstrates the influence of the family environment on the formation of eating habits, highlighting the role of parents, food availability in the home, and family mealtime dynamics [8].

### 1.2. The Impact of the Digital Environment

Intensive screen use from early childhood is consistently associated with lower levels of physical activity, increased sedentary behaviour, sleep disturbances, and a higher risk of cardiometabolic and emotional disorders [9,10,11]. These findings have driven growing interest in understanding the effects of screen time and in designing effective intervention strategies within digital contexts.

### 1.3. Innovative Interventions in School, Family, and Digital Settings

Multicomponent school-based interventions continue to demonstrate effectiveness in promoting improvements in health-related behaviours, particularly when they involve modifications to the food environment and active engagement of the educational community [12,13]. At the same time, there has been significant growth in interventions based on mobile technologies, digital applications, and e-Health platforms, which show considerable potential to facilitate sustained behaviour change among children and adolescents [14,15,16].

### 1.4. New Markers for Early Detection of Obesity Risk

Changes in BMI trajectories and dietary quality indices provide new opportunities for the early identification of cardiometabolic risk [17,18]. In parallel, compositional analyses of time allocated to physical activity, sleep, and sedentary behaviours are enabling a more precise understanding of how these behaviours interact and contribute to obesity risk [19].

## 2. Existing Knowledge Gaps

Despite these advances, important gaps in knowledge remain. Deeper inquiry into the interaction of multiple behaviours, adaptation to sociocultural diversity and emerging environments, and the need for more long-term studies remain pressing issues.

Although evidence exists regarding the roles of diet, physical activity, sleep, and screen time, further understanding is needed into how these behaviours interact and which combinations are most influential for health and obesity risk [19]. Cultural and socioeconomic differences shape the effectiveness of interventions, underscoring the need to tailor strategies to local contexts and to reduce health inequalities [20]. Digital marketing targeted at children, the increasing availability of ultra-processed foods, and shifts in food environments continue to present significant challenges for child health [21,22]. While many interventions show immediate benefits, their long-term sustainability remains limited or insufficiently documented. More longitudinal research is needed, spanning the transition into late adolescence and adulthood [23].

## 3. Contribution of This Special Issue

This Special Issue makes a significant contribution to addressing these gaps by presenting studies that examine dietary behaviours and their relationship with excess weight; deepen the analysis of interactions between screen time, sleep, physical activity, and well-being; evaluate digital interventions aimed at promoting healthy habits among young people; explore the roles of family, school, and community settings as intervention environments; and introduce new approaches for the early detection of cardiometabolic risk.

The articles included provide an updated and multidimensional perspective on the determinants of child and adolescent overweight and obesity, offering relevant evidence for future research and the development of public health policies.

## 4. Key Directions for Future Research

Models that jointly integrate diet, sleep, physical activity, sedentary behaviour, and mental health will be essential for capturing the complexity of human behaviour in early life [24].

Digital tools will enable the development of interventions tailored to the needs of individuals and families, increasing their feasibility, adherence, and scalability [25].

Scientific advances should support the design and evaluation of public policies aimed at promoting healthier food environments, regulating advertising directed at children, and implementing integrated strategies in schools and municipalities [26].

It is crucial to promote longitudinal and implementation studies that assess the durability of behavioural changes and their contribution to health across the life course [27].

## References

[1] Stiglic N., Viner R.M. (2019). Effects of screentime on the health and well-being of children and adolescents: A systematic review of reviews. BMJ Open.

[2] World Health Organization (2025). Obesity and Overweight. https://www.who.int/news-room/fact-sheets/detail/obesity-and-overweight.

[3] Wilhite K., Booker B., Huang B.H., Antczak D., Corbett L., Parker P., Noetel M., Rissel C., Lonsdale C., Del Pozo Cruz B. (2023). Combinations of Physical Activity, Sedentary Behavior, and Sleep Duration and Their Associations with Physical, Psychological, and Educational Outcomes in Children and Adolescents: A Systematic Review. Am. J. Epidemiol..

[4] Jia P., Shi Y., Jiang Q., Dai S., Yu B., Yang S., Qiu G., Yang S. (2023). Environmental determinants of childhood obesity: A meta-analysis. Lancet Glob. Health.

[5] Maia C., Braz D., Fernandes H.M., Sarmento H., Machado-Rodrigues A.M. (2025). The Impact of Parental Behaviors on Children’s Lifestyle, Dietary Habits, Screen Time, Sleep Patterns, Mental Health, and BMI: A Scoping Review. Children.

[6] Ricotti R., Caputo M., Monzani A., Pigni S., Antoniotti V., Bellone S., Prodam F. (2021). Breakfast Skipping, Weight, Cardiometabolic Risk, and Nutrition Quality in Children and Adolescents: A Systematic Review of Randomized Controlled and Intervention Longitudinal Trials. Nutrients.

[7] Monteiro C.A., Cannon G., Moubarac J.C., Levy R.B., Louzada M.L.C., Jaime P.C. (2018). The UN Decade of Nutrition, the NOVA food classification and the trouble with ultra-processing. Public Health Nutr..

[8] Patrick H., Nicklas T.A. (2005). A review of family and social determinants of children’s eating patterns and diet quality. J. Am. Coll. Nutr..

[9] Robinson T.N., Banda J.A., Hale L., Lu A.S., Fleming-Milici F., Calvert S.L., Wartella E. (2017). Screen Media Exposure and Obesity in Children and Adolescents. Pediatrics.

[10] Przybylski A.K., Weinstein N. (2019). Digital Screen Time Limits and Young Children’s Psychological Well-Being: Evidence from a Population-Based Study. Child Dev..

[11] Chaput J.P., Gray C.E., Poitras V.J., Carson V., Gruber R., Olds T., Weiss S.K., Connor Gorber S., Kho M.E., Sampson M. (2016). Systematic review of the relationships between sleep duration and health indicators in school-aged children and youth. Appl. Physiol. Nutr. Metab..

[12] Langford R., Bonell C.P., Jones H.E., Pouliou T., Murphy S.M., Waters E., Komro K.A., Gibbs L.F., Magnus D., Campbell R. (2014). The WHO Health Promoting School framework for improving the health and well-being of students and their academic achievement. Cochrane Database Syst. Rev..

[13] Micha R., Karageorgou D., Bakogianni I., Trichia E., Whitsel L.P., Story M., Mozaffarian D. (2018). Effectiveness of school food environment policies on children’s dietary behaviors: A systematic review and meta-analysis. PLoS ONE.

[14] Azevedo L.B., Stephenson J., Ells L., Adu-Ntiamoah S., DeSmet A., Giles E.L., Haste A., O’Malley C., Jones D., Chai L.K. (2022). The effectiveness of e-health interventions for the treatment of overweight or obesity in children and adolescents: A systematic review and meta-analysis. Obes Rev..

[15] Cushing C.C., Steele R.G. (2011). A meta-analytic review of eHealth interventions for pediatric obesity prevention. Prev. Med..

[16] Riazi N., Ramanathan S., O’Neill M., Tremblay M.S., Faulkner G. (2022). The effectiveness of wearable activity trackers for increasing physical activity among children and adolescents: A systematic review and meta-analysis. Obes. Rev..

[17] Rolland-Cachera M.F. (2011). Childhood obesity: Current definitions and recommendations for their use. Int. J. Pediatr. Obes..

[18] Buscot M.J., Thomson R.J., Juonala M., Sabin M.A., Magnussen C.G., Raitakari O.T. (2020). BMI trajectories associated with adolescent adiposity rebound and their cardiometabolic outcomes. J. Clin. Endocrinol. Metab..

[19] Dumuid D., Stanford T.E., Martin-Fernández J.A., Pedišić Ž., Maher C.A., Lewis L.K., Olds T., Katzmarzyk P.T. (2020). Compositional data analysis for physical activity, sedentary time and sleep research. Stat. Methods Med. Res..

[20] Swinburn B.A., Sacks G., Hall K.D., McPherson K., Finegood D.T., Moodie M.L., Gortmaker S.L. (2011). The global obesity pandemic: Shaped by global drivers and local environments. Lancet.

[21] Kelly B., Vandevijvere S., Freeman B., Jenkin G. (2015). New media but same old tricks: Food marketing to children in the digital age. Curr. Obes. Rep..

[22] Swinburn B., Kraak V.I., Rutter H., Vandevijvere S., Lobstein T., Sacks G., Gomes F., Marsh T., Magnusson R. (2015). Strengthening accountability systems to create healthy food environments and reduce global obesity. Lancet.

[23] Brown T., Moore T.H., Hooper L., Gao Y., Zayegh A., Ijaz S., Elwenspoek M., Foxen S.C., Magee L., O’Malley C. (2019). Interventions for preventing obesity in children. Cochrane Database Syst. Rev..

[24] Walsh J.J., Barnes J.D., Cameron J.D., Goldfield G.S., Chaput J.P., Gunnell K.E., Ledoux A.A., Zemek R.L., Tremblay M.S. (2018). Associations between 24 hour movement behaviours and global cognition in US children: A cross-sectional observational study. Lancet Child Adolesc. Health.

[25] Patrick K., Hekler E.B., Estrin D., Mohr D., Riper H., Crane D., Godino J.G., Riley W.T. (2016). The pace of technologic change: Implications for digital health behavior intervention research. Am. J. Prev. Med..

[26] Hawkes C., Smith T.G., Jewell J., Wardle J., Hammond R.A., Friel S., Thow A.M., Kain J. (2015). Smart food policies for obesity prevention. Lancet.

[27] Singh A.S., Mulder C., Twisk J.W.R., van Mechelen W., Chinapaw M.J.M. (2008). Tracking of childhood overweight into adulthood: A systematic review. Obes. Rev..

